# Central Role of Cellular Senescence in TSLP-Induced Airway Remodeling in Asthma

**DOI:** 10.1371/journal.pone.0077795

**Published:** 2013-10-22

**Authors:** Jinxiang Wu, Fangzheng Dong, Rui-An Wang, Junfei Wang, Jiping Zhao, Mengmeng Yang, Wenbin Gong, Rutao Cui, Liang Dong

**Affiliations:** 1 Department of Respiratory, Qilu Hospital of Shandong University, Jinan, Shandong, China; 2 University of Iowa College of Liberal Arts and Sciences, Iowa City, Iowa, United States of America; 3 Department of Pathology, Fourth Military Medical University, Xian, Shanxi, China; 4 Longhua Hospital, Shanghai University of TCM, Shanghai, China; 5 Department of Dermatology & Biochemistry, Boston University School of Medicine, Boston, Massachusetts, United States of America; Cedars-Sinai Medical Center, United States of America

## Abstract

**Background:**

Airway remodeling is a repair process that occurs after injury resulting in increased airway hyper-responsiveness in asthma. Thymic stromal lymphopoietin (TSLP), a vital cytokine, plays a critical role in orchestrating, perpetuating and amplifying the inflammatory response in asthma. TSLP is also a critical factor in airway remodeling in asthma.

**Objectives:**

To examine the role of TSLP-induced cellular senescence in airway remodeling of asthma *in* *vitro* and *in* *vivo*.

**Methods:**

Cellular senescence and airway remodeling were examined in lung specimens from patients with asthma using immunohischemical analysis. Both small molecule and shRNA approaches that target the senescent signaling pathways were used to explore the role of cellular senescence in TSLP-induced airway remodeling *in* *vitro*. Senescence-Associated β-galactosidase (SA-β-Gal) staining, and BrdU assays were used to detect cellular senescence. In addition, the Stat3-targeted inhibitor, WP1066, was evaluated in an asthma mouse model to determine if inhibiting cellular senescence influences airway remodeling in asthma.

**Results:**

Activation of cellular senescence as evidenced by checkpoint activation and cell cycle arrest was detected in airway epithelia samples from patients with asthma. Furthermore, TSLP-induced cellular senescence was required for airway remodeling *in* *vitro*. In addition, a mouse asthma model indicates that inhibiting cellular senescence blocks airway remodeling and relieves airway resistance.

**Conclusion:**

TSLP stimulation can induce cellular senescence during airway remodeling in asthma. Inhibiting the signaling pathways of cellular senescence overcomes TSLP-induced airway remodeling.

## Introduction

 Asthma is characterized by chronic inflammation and structural alterations in the airways [1] and airway remodeling is a common feature of asthma [2]. Remodeling is caused by repetitive injury to the airway wall arising from cycles of inflammation and repair [3]. Histological characteristics of airway remodeling include thickening of the lamina reticularis, epithelial shedding, subepithelial fibrosis, inflammatory cell infiltration, goblet cell hyperplasia, myofibroblast proliferation, smooth muscle hyperplasia and hypertrophy, and neovascularization of the airway wall [4]. These features and airway hyper-responsiveness (AHR) are common in severe lung diseases such as asthma [5].

 Airway epithelia produce some inflammatory factors that play critical roles in regulating metabolic and immunologic responses within airways. These inflammatory factors are often involved in epithelial damage in patients with asthma [6]. TSLP, an IL-7-like inflammatory factor [7], is secreted by bronchial and lung epithelial cells [8–10]. Several groups have demonstrated that TSLP levels are increased in asthmatic epithelia [11,12]. TSLP protein levels are also increased in the airway epithelia and bronchoalveolar lavage fluids (BALF) collected from patients with asthma [8,13]. Recently, several independent genome-wide association studies have demonstrated that TSLP is a susceptibility locus for asthma [14,15]. 

 Senescent cells are frequently found at sites of chronic disease. Many older patients have histological and symptomatic features of chronic obstructive pulmonary disease (COPD) and asthma [16,17]. Alveolar and airway cell senescence from cigarette smoke and aging contributes to the destruction of alveoli by limiting the proliferative capacity necessary for tissue repair and promoting chronic inflammation. Both of these processes are hallmarks of COPD [18]. In addition, cigarette smoke can induce expression of senescence markers, such as SA-β-gal in lung epithelial cells and fibroblasts [19,20]. Certain cytokines are crucial in airway remodeling and cellular senescence. For example, paracrine IL-6 and IL-8 both induce maintenance of the senescent phenotype [18,21–23].

To determine whether inhibiting cytokine-induced cellular senescence can overcome airway remodeling in asthma, we explored the role of senescence in TSLP-induced airway remodeling in asthma *in vitro* and *in vivo*. We found that 1) senescent signaling pathways are activated in airway epithelium in patients with asthma, 2) TSLP stimulation induced cellular senescence and silencing of cellular senescence pathways inhibits TSLP-induced airway remodeling *in vitro* and 3) Stat3-targeted therapies overcome TSLP-induced airway remodeling by inhibiting cellular senescence *in vitro* and *in vivo*. 

## Materials and Methods

### Ethics Statement

The study was approved by the Ethics Review Committee for Human Studies at Qilu Hospital, Shandong University. Each subject provided written, informed consent. Animal experiments were performed in accordance with the Institutional Animal Care and Use Committee of Shandong University.

The protocol was approved by the Committee on the Ethics of Animal Experiments of Shandong University. Each mouse was anesthetized with intraperitoneal ketamine (35 mg/kg). Cervical dislocation was used to euthanize mice. All efforts were made to minimize pain and discomfort. 

### Patients

The study was approved by the Ethics Review Committee for Human Studies at Qilu Hospital, Shandong University. Each subject provided written, informed consent. Endobronchial biopsies from 10 asthma patients were obtained from Shandong Provincial Chest Hospital (Jinan, Shandong, China). Healthy control specimens were obtained from Qilu Hospital of Shandong University (Jinan, Shandong, China), and were from pneumoresections. Subject characteristics are shown in Table 1.

**Table 1:** Characteristics of healthy control subjects and asthmatic patients.

	**Healthy control subjects**	**Asthmatic patients**
**Sex (M/F)**	5/5	6/4
**Age (y)**	27.2 ± 5.31	29.7 ±4.14
**FEV1(L)**	4.17 ±0.69	3.68 ± 0.84
**FEV1(%)**	99.6 ± 7.15	91.8 ± 19.85
**PC20(mg/mL)**	145.17±20.12	1.99 ±0.84

Values are presented as means ± SDs.

### Histological analysis

Immunohistochemical staining was performed as described [24]. The immunohistochemical scoring (H score) was determined by multiplying the staining intensity by the percentage of positive tumor cells [25,26]. These primary antibodies were used: anti-p16 （f-12, SC-1661）, anti-p21（C-19, SC397, anti-TSLP (M-20, SC19178), anti-α-smooth muscle actin (α-SMA) (1A4, ab7817), anti-collagen I (ab34710) and anti-Ki67(M-19, SC7846). All these antibodies were purchased from Santa Cruz Bio-technology, Inc. (Santa Cruz, CA, USA) or Abcam Inc. (Cambridge, MA, USA). 

### Cells and regents

The human bronchial epithelial cell line, BEAS-2B, was derived from human bronchial epithelia and was purchased from ATCC (Manassas, VA). TSLP was purchased from R&D Systems (Minneapolis, MN, USA). WP1066 was purchased from Santa Cruz Bio-technology, Inc. (Santa Cruz, CA, USA). 

### Staining for SA-β-Galactosidase

BEAS-2B cells were fixed in 2% formaldehyde and 0.2% glutaraldehyde for 10 minutes at room temperature, washed in PBS, and incubated for 24 h at 37 °C with SA-β-Gal (Sigma-Aldrich, St. Louis, MO, USA) in staining solution (40 mM sodium citrate (pH 6), 150 mM, NaCl, 5 mM potassium ferricyanide, 5 mM potassium ferrocyanide, 2 mM MgCl_2_ and 1 mg/ml bromo-4-chloro-3-indolyl-β-D-galactoside). 

### BrdU Analysis

BrdU Cell Proliferation Assay Kit was purchased from Chemicon International (Temecula, CA,USA) and used following manufacturer’s protocol. 

### Western Blotting

These primary antibodies were used: anti-p16 (f-12, SC-1661), anti-p21(C-19, SC397), anti-TSLP (M-20, SC19178), anti-α-SMA (1A4, ab7817), anti-collagen I (ab34710) and α – tubulin (B3, ab11324). All these antibodies were purchased from Santa Cruz Bio-technology, Inc. (Santa Cruz, CA, USA) or Abcam Inc. (Cambridge, MA, USA). 

### MTT (3-(4,5-Dimethylthiazol-2-yl)-2,5-Diphen​yltetrazoliumBromide) assay

BEAS-2B cells plated in 96-well plates were treated with TSLP. Cell viability was assayed using the Vibrant® MTT Cell Proliferation Assay Kit (Molecular Probes) following the manufacturer’s protocol. 

### ELISA

TSLP Quantikine Elisa kit was purchased from R&D systems, and we carried out the protocol according to the manual instruction.

### Immunofluorescence microscopy

BEAS-2B cells plated on microscope cover slides were treated for 24 h with 1.5 ng/ml TSLP. Cells to be stained with anti-Ki67 were fixed with 2% paraformaldehyde in PBS for 30 min at RT and permeabilized by Triton X-100 (0.2% in PBS with 1% BSA) for 15 min at RT. Samples were blocked using TBS/0.05% Tween-20 with 3% skim milk for 1 h at RT. The mouse anti- Ki67 antibody was diluted 1:200 in TBS/0.05% Tween-20 and applied to cover slides with incubation overnight at 4°C. After washing with TBS/0.05% Tween-20, the cover slides were incubated with secondary Rabbit F(ab')2 Anti-Goat IgG H&L (Alexa Fluor® 488) (ab169344) diluted 1:00 in TBS/0.05% Tween-20 for 2 h at RT and washed. DAPI was used to stain the nuclei.

### Short Hairpin RNAs

Short hairpin RNA duplexes that target p21and p16 were purchased from Open Biosystem (Huntsville, AL, 35806). The specificity of these shRNAs have been evaluated previously [27]. 

### Mouse Asthma Model and WP1066 Treatment

BALB/c mice were purchased from the Center of Experimental Animals of Shandong University School of Medicine and animal experiments were performed in accordance with the Institutional Animal Care and Use Committee of Shandong University.

Mice (female, 6-week-old, 18 - 20g each) were either control, untreated, OVA (Sigma-Aldrich, St. Louis, MO)-induced asthma model or WP1066-treated (n=10). Mice were sensitized with OVA as described previously [28]. WP1066-treatment mice were treated once daily with 40mg/kg WP1066 by intraperitoneal injection 1h before the OVA challenge. Mice were injected with saline, OVA or OVA+WP1066 during airway challenge. Mice were then examined for airway remodeling 48h after the last challenge and lung tissues were collected for immunohistochemistry analysis.

### Analysis of Airway Hyper responsiveness (AHR)

Methacholine was used at 0, 3, 6, 9, 12 g/L and a dose-response curve was constructed. Measurements of AHR were made 1 min after each dose and 2 min between doses. Results are expressed as the maximum resistance after each dose minus baseline (PBS alone) resistance.

### Statistical analysis

Statistical analysis was performed using the SPSS 12.0 software package. Data were analyzed using Student’s *t*–test. Significance was defined for *p*<0.05.

## Results

### p16 and p21 protein levels are upregulated in airway epithelium of patients with asthma

**Figure 1:**
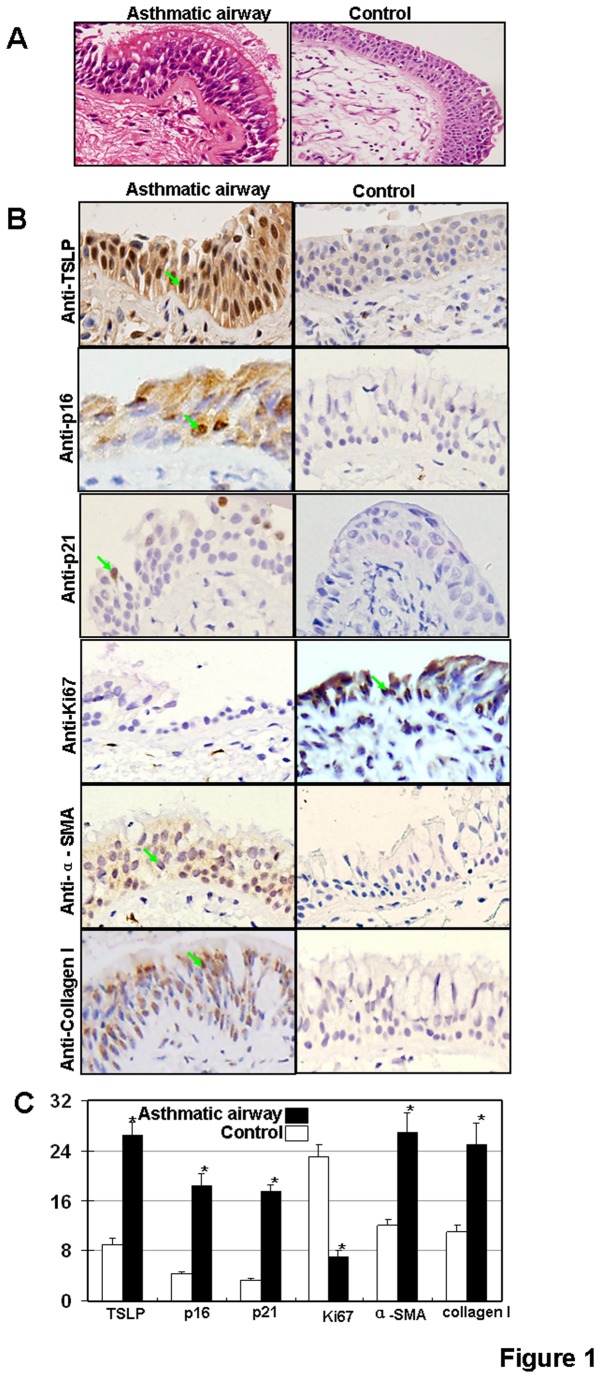
Protein expressions of p16 and p21 in human asthmatic airway epithelium tissue. (**A**) Micrographs of histological sections of the asthmatic human airway showing the loss of epithelial integrity and wall thickening by H and E staining. Magnification 400X. (**B**) TSLP, p16, p21, Ki67, α-SMA and collagen I expression. Arrows indicate areas of positive expression. Magnification, 400×. (**C**) Bimodal H score distribution of TSLP, p16, p21, Ki67, α-SMA and collagen I immunoperoxidase reactions.

Cellular senescence is a state of irreversible growth arrest and is induced by a wide variety of conditions, including telomere shortening (replicative senescence) and telomere-independent signals (stress-induced senescence) [29,30]. Previous reports have demonstrated that cigarette smoke induces airway epithelial cellular senescence [20,21] and senescent cells are readily detected in airway epithelia of patients with COPD [31]. Given the common features of asthma and COPD, specifically, airway remodeling, we examined cellular senescence in airway remodeling in asthma. We first asked if senescent signaling pathways are activated in airway epithelia of asthma. Two major signaling pathways are involved in senescence: the p19^ARF^/p53/p21 pathway and the p16^INK4^/CDK/pRb pathway [32]. Several groups have found that p21 mRNA and protein levels are increased in asthma airways, and expression levels correlate with asthma severity [33–35]. In addition, thioredoxin (TRX) can repress the expression of p21 and block airway remodeling in an asthma mouse model [36]. We examined p21 and p16 protein levels in bronchial epithelia collected from patients with asthma and found increases in p16 and p21 protein levels in epithelial cells from asthma patients (Figure 1B & C). To determine if upregulation of p16 and p21 correlates with lower levels of proliferation, we examined the expression of Ki67 in asthma epithelia samples. As expected, the expression of Ki67 protein was repressed in epithelia samples from asthma patients (Figure 1B & C). To examine whether senescent pathway activation correlates with airway remodeling, we examined markers of airway remodeling in asthma patient samples by looking at collagen I and α-SMA protein levels. As expected, increased expression levels of collagen I were found in asthma patient epithelia (Figure 1B & C). In addition, α-SMA was highly expressed in the basement membrane of thickened airway walls of asthma samples (Figure 1B & C). These results suggest that pathways of cellular senescence and airway remodeling are activated in airway epithelia from patients with asthma.

### Cellular senescence is induced by TSLP in human airway epithelial cells *in vitro*

**Figure 2:**
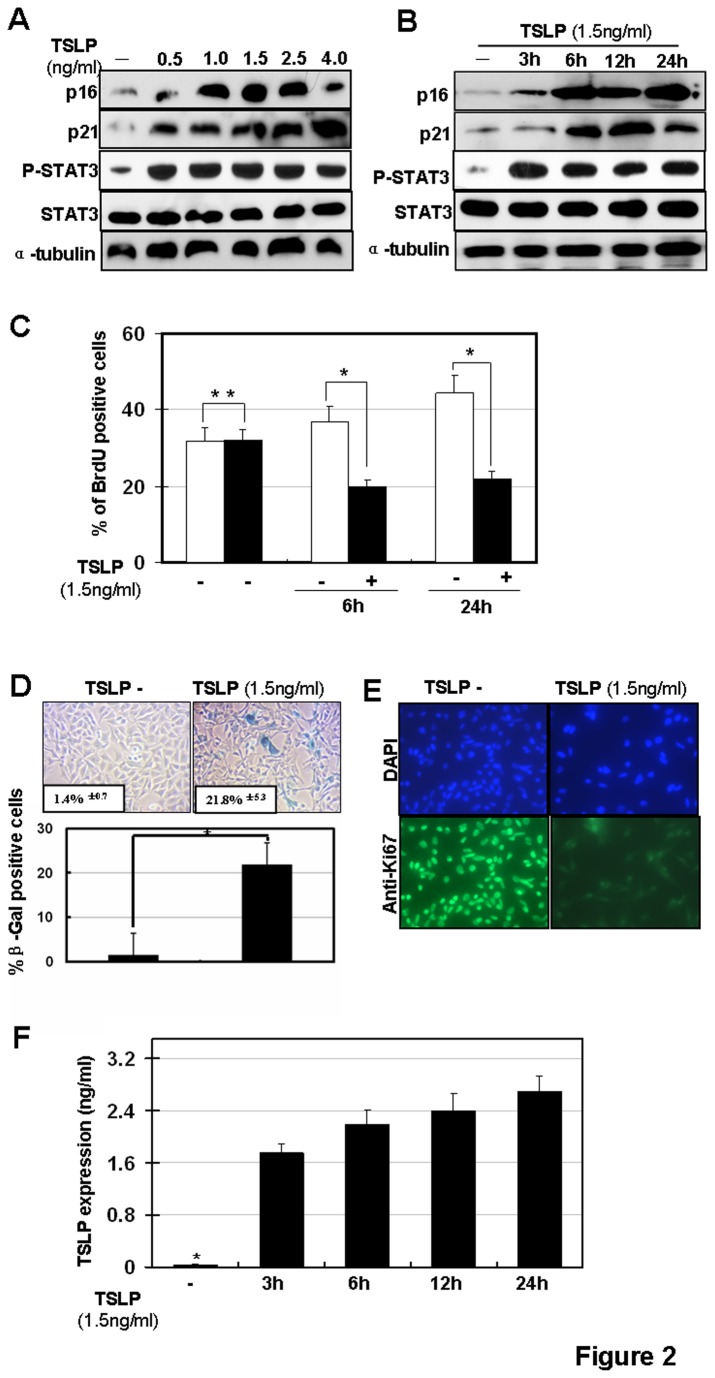
Cellular senescence is induced by TSLP stimulation *in* *vitro*. (**A**) TSLP-induced p16 and p21 upregulation occurs in a TSLP dose-dependent manner in human bronchial epithelial BEAS-2B cells. BEAS-2B cells were stimulated with different doses of TSLP as indicated for 6h. Protein expressions of p16, p21 and phospho-Stat3 (Try705) were detected by western blotting. (**B**) BEAS-2B cells were stimulated by 1.5ng/ml TSLP and protein expressions of p16 and p21 were detected by western blotting. (**C**) BEAS-2B cells were stimulated with 1.5ng/ml TSLP then stained for BrdU. (*p< 0.05). BEAS-2B cells were stimulated with 1.5ng/ml TSLP then stained for SA-β-gal activity at 6 and 24 hours post stimulation. (**D**) upper panel: SA-β-gal staining; lower panel: quantification of SA-β-gal positive cells. (*p < 0.05); (**E**) Ki67 staining. (**F**) Levels of TSLP in culture media were examined by ELISA.

To further explore the role of cellular senescence in airway remodeling of asthma, we tested whether TSLP, a critical cytokine in airway remodeling in asthma, induces senescence in human airway epithelial cells. Previous studies demonstrated that the neutralization of TSLP could inhibit airway remodeling in a murine model of allergic asthma induced by dust mites [37]. In our experiment, the airway epithelial cell line, BEAS-2B, was used [38].. BEAS-2B cells were treated with TSLP (1.5ng/ml) and SA-β-gal staining was performed to detect senescent cells. β-galactosidase, the lysosomal hydrolase, is active at pH 4, but SA-β-gal is active at pH 6 and is only present in senescent cells; allowing the two activities to be readily distinguished [39]. Phospho-Stat3, a downstream target of TSLP [40] served as a control to monitor the activation of TSLP signaling in BEAS-2B cells. We found that cellular senescence was induced by TSLP stimulation. Specifically, senescent cells increased >15 fold upon treatment with 1.5 ng/ml TSLP (Figure 2A). To confirm these findings, p16 and p21 expression analysis and BrdU labeling were also performed. As shown in Figure 2B, upregulation of p16 and p21 was detected from 3-24 hours after TSLP stimulation (Figure 2B). TSLP-induced p16 and p21 upregulation occurred in a TSLP dose-dependent manner (Figure 2A). Furthermore, cell proliferation analysis (BrdU labeling) revealed that TSLP stimulation significantly inhibited cell proliferation up to 24 hours after TSLP treatment (Figure 2C). In addition, there was increased expression of SA-β-gal and decreased Ki67 expression in BESA-2B cells upon TSLP treatment. (Figure 2D & E). Furthermore, we also found TSLP stimulation induced TSLP secretion (Figure 2F). Taken together, these results indicate that TSLP stimulation induces cellular senescence in BEAS-2B cells. 

### Cellular senescence induction is required in TSLP-induced airway remodeling

**Figure 3:**
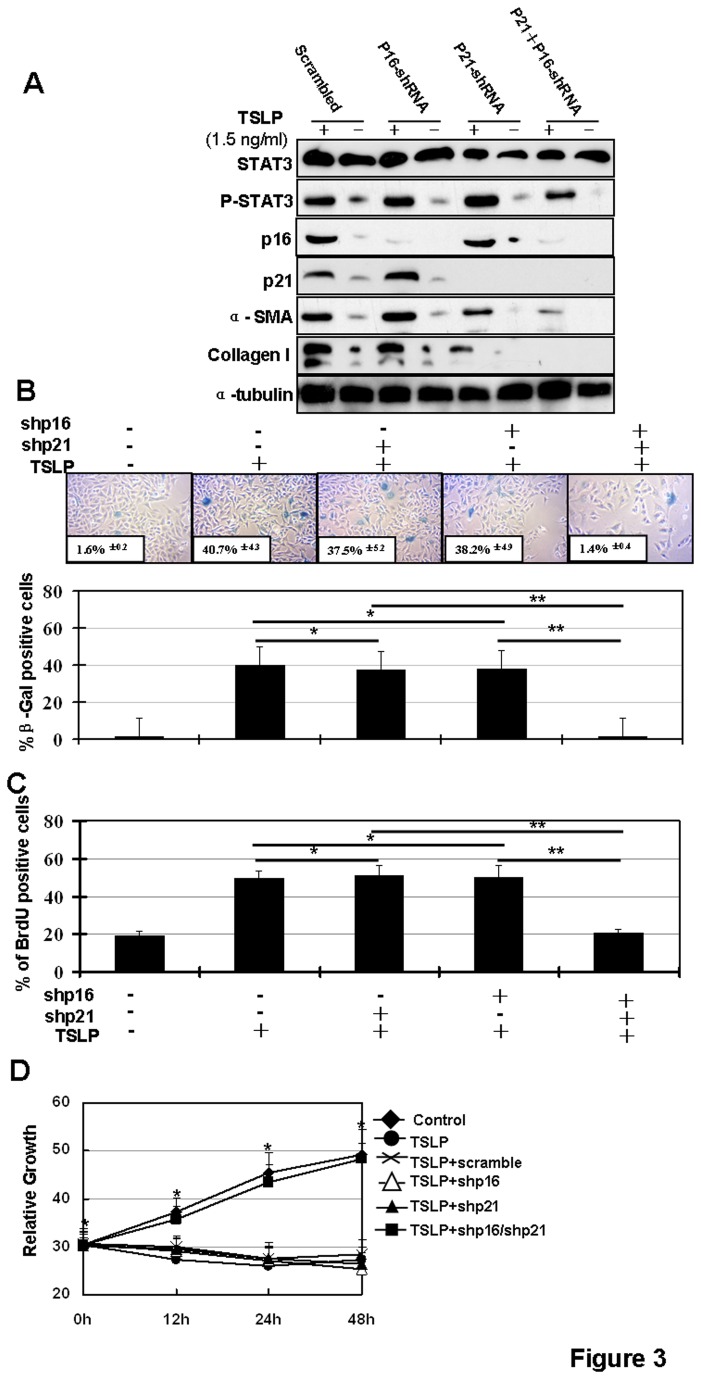
Senescent inhibition overcomes TSLP-induced airway remodeling in vitro. BEAS-2B cells with stable shp16, shp21 or both were incubated with TSLP (1.5ng/ml) for 6 h. (**A**) Cells were collected and total proteins were extracted and analyzed by western blotting. (**B**) Cells were fixed and stained with SA-β-gal (upper panel) and then positive SA-β-gal cells were quantified (*p>0.05, **p < 0.05) (low panel). (**C**) Cells were stained with BrdU (*p>0.05, **p < 0.05). (**D**) Senescent inhibition overcomes TSLP-induced cell growth inhibition in vitro. The relative cell number was detected to evaluate cell growth at different time points using MTT assays.

We examined the role of cellular senescence in airway remodeling in asthma. For this analysis, we generated silencing cell lines of BEAS-2B cells by stably expressing shp16, and/or shp21. It has been shown that silencing of p16 and p21 signaling pathways together inhibits cellular senescence [41]. We examined TSLP-activated airway remodeling in p16 and p21 silenced cells (Figure 3A). We found that TSLP stimulation induces the activation of airway remodeling markers, including collagen I and α-SMA [24]. This activation is inhibited when p16 and p21 are both silenced. As expected, silencing the p16 or p21 pathways alone does not inhibit TSLP-induced activation of airway remodeling *in vitro* (Figure 3A). To examine if both of p16 and p21 silencing can inhibit TSLP-induced cellular senescence, SA-β-gal expression analysis was performed and cell proliferation was tested using BrdU labeling and MTT analysis in TSLP-stimulated BEAS-2B cells with stable p16 and/or p21 silencing vectors. As expected, silencing of both p16 and p21 pathways inhibits TSLP-induced SA-β-gal activation (Figure 3B) and inhibits cell proliferation (Figure 3C & D). These results suggest that cellular senescence is required in TSLP-activated airway remodeling. 

### A Stat3 inhibitor suppresses senescence-associated airway remodeling in BEAS-2B cells

**Figure 4:**
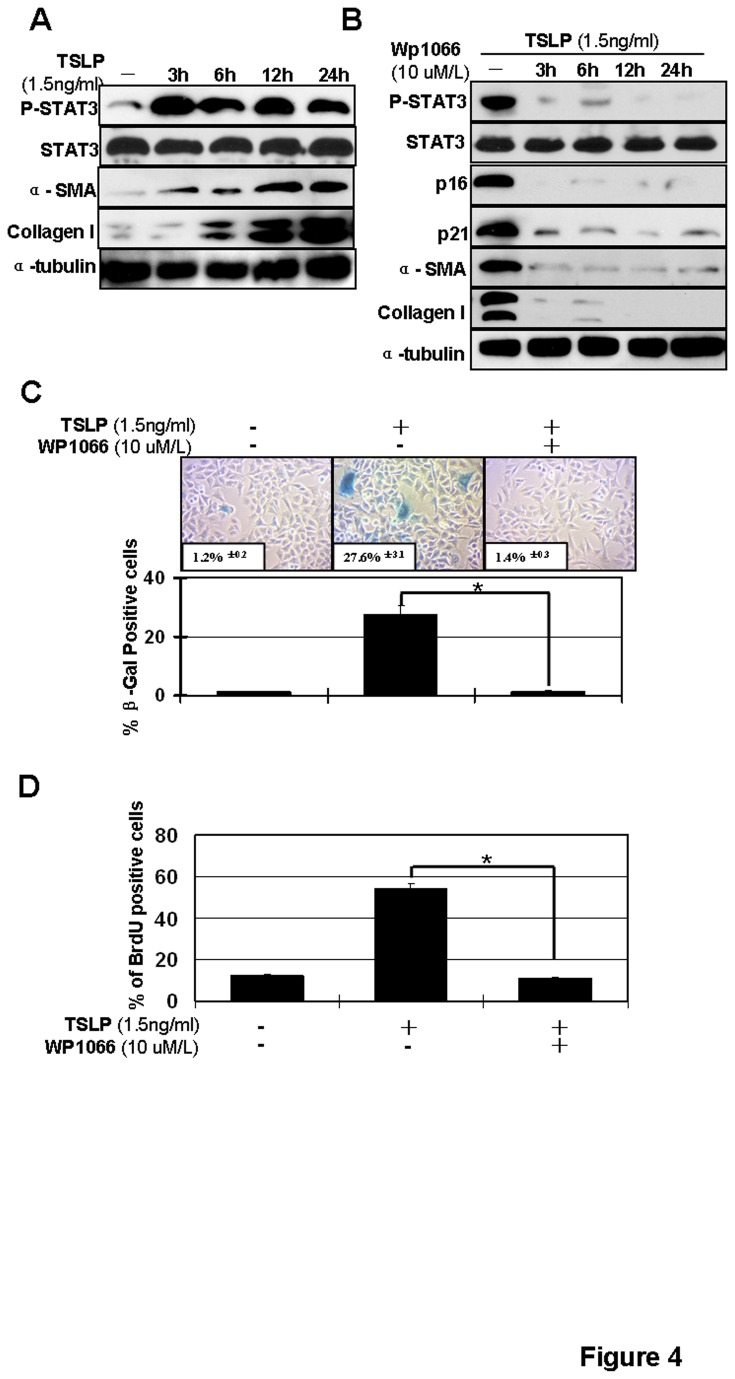
Inhibition of Stat3 overcomes TSLP-induced senescence and airway remodeling in BESA-2B cells. (**A**) TSLP-induced activation of Stat3 and airway remodeling. BESA-2B cells were stimulated with 1.5ng/ml TSLP and total protein was collected at different time points. Protein expressions of phospho-Stat3, Stat3, α-SMA and Collagen I were analyzed by western blotting along with α-tubulin, which serves as a loading control. BESA-2B cells were stimulated with 1.5ng/ml TSLP and 10μM WP1066 as indicated. (**B**) Total protein was collected after 6 hour TSLP stimulation. Protein expressions of phospho-Stat3, Stat3, p21, p16, α-SMA and Collagen I were analyzed by western blotting. Expression of α-tubulin, serves as a loading control. (**C**) Cells were fixed and then stained with SA-β-gal (upper panel) and SA-β-gal positive cells were quantified (*p < 0.05) (low panel). (**D**) Cells were stained with BrdU (*p < 0.05).

Previously, we demonstrated that exogenous TSLP activated the Stat3 signaling pathway in human lung fibroblasts [24] and we confirm these data here (Figure 4A). To further examine the involvement of Stat3 in TSLP-induced senescence in BEAS-2B cells, BEAS-2B cells were incubated with 10μM of the Stat3 inhibitor WP1066 for 2h and then treated with different concentrations of TSLP. Then, SA-β-gal, p21 and p16 expression and BrdU labeling analyses were performed. Collagen I and α–SMA expression were used to monitor airway remodeling. We found that WP1066 preincubation suppressed TSLP-induced senescence and airway remodeling in BEAS-2B cells (Figure 4B & C & D).

### WP1066 treatment attenuates airway hyper-responsiveness (AHR) and airway remodeling in a mouse asthma model

**Figure 5:**
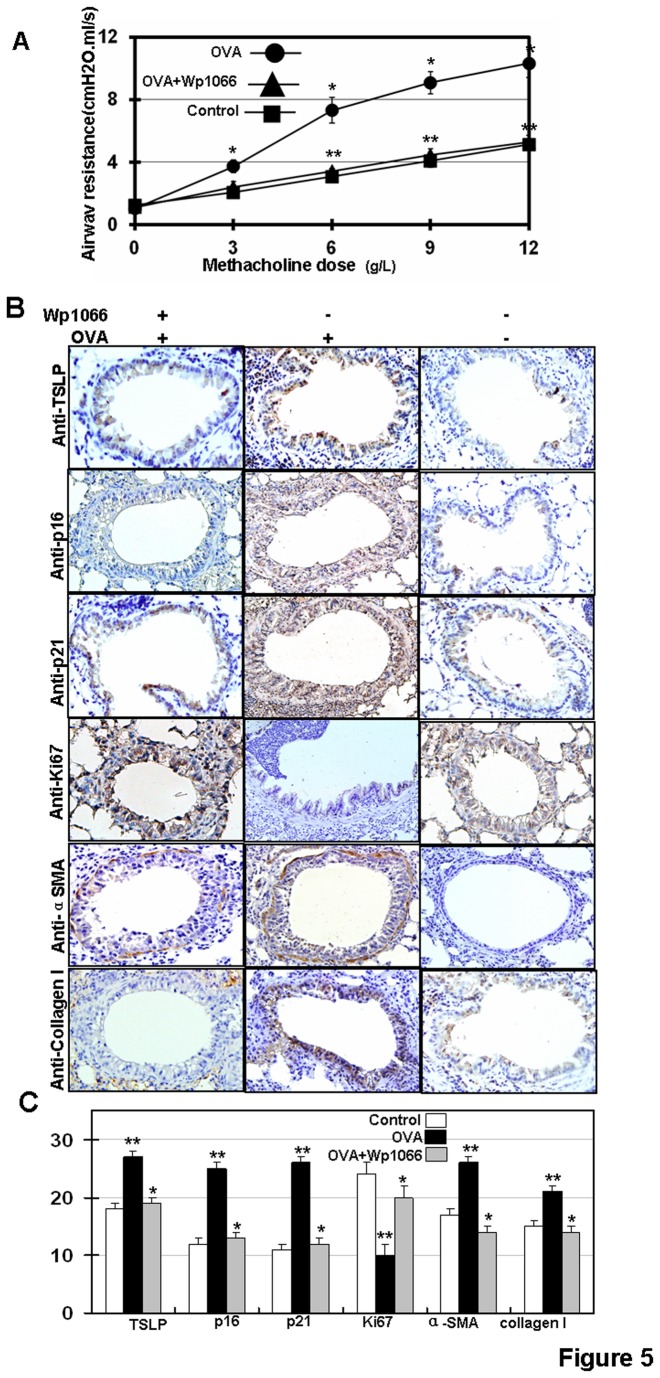
Experimental therapies with WP1066 in OVA-challenged chronic asthmatic mice. (**A**) OVA-challenge was performed in BALB/c mice as described [28]. WP1066 was administered by intraperitoneal injection at doses of 40mg/kg 1h before the OVA-challenge. Airway resistance was measured using increasing concentration of methacholine and assessed using the flexiVent system. Results are expressed as the mean of experiments done in triplicate ± the standard error of the mean (SEM) (* p<0.05, **p>0.05 *vs* *control*). (**B**) TSLP, p16, p21, Ki-67, α-SMA and collagen I protein expressions were analyzed, 200×. (**C**) Bimodal H score distribution of TSLP, p16, p21, Ki67, α-SMA and collagen I immunoperoxidase reactions are presented.

To determine whether WP1066 treatment can relieve airway resistance *in vivo*, we generated a mouse asthma model by daily OVA challenge. Airway resistance was monitored using flexiVent. We found that WP1066 treatment significantly overcame airway resistance (Figure 5A). We also found that OVA-challenge activated cellular senescence in the mouse airway epithelium (Figure 5B & C). In addition, OVA-challenge induced the expression of α-SMA and collagen I in mouse airway epithelia (Figure 5B & C. These results indicate that chronic allergen exposure in mice promotes bronchial remodeling. In addition, we used p21, p16 and Ki67 as senescence markers in airway epithelium *in vivo*. We found that inhibiting Stat3 by WP1066 inhibited cellular senescence and resulted in the upregulation of α-SMA and collagen I in mouse airway epithelia (Figure 5B & C). 

## Discussion

### Cellular senescence in airway remodeling of asthma

Senescence is an important fail-safe mechanism often arising in response to telomere erosion and stress. Chronic inflammation is a major histological characteristic of asthma and plays a critical role in airway remodeling. Chronic inflammation also represents tissue stress. Therefore, it is not surprising that cellular senescence is induced in airway epithelia of asthma. Senescence arises through checkpoint activation and cell cycle arrest and is a barrier to cell proliferation and tumorgenesis [42]. In fact, activation of cell cycle checkpoint proteins prevents the replication of genomically unstable cells [43]. Previous reports have demonstrated the critical role of cellular senescence in COPD and other respiratory diseases, such as pulmonary fibrosis and lung cancer [44–46]. Here we further demonstrate that cellular senescence is required in TSLP-induced of airway remodeling in asthma. We confirm the upregulations of p16 and p21 and the repression of Ki67 in epithelia from asthma patients (Figure 1). We also found that upregulation of p16 and p21 is required in TSLP-induced airway remodeling (Figure 3). Both the p16 and p21 pathways are involved in senescence [32]. Upregulation of p21 in asthma has been reported. Furthermore, previous reports demonstrate that the increased levels of p21 protein correlates with asthma severity [33–35]. The mechanism of p21 activation in asthma has been investigated and evidence indicates that some cytokines induce the expression of p21. *In vivo* studies demonstrate the therapeutic potential of p21-targeted therapy in asthma. For example, thioredoxin (TRX) reduces gene expression of TGF-β1, EGFR, and p21 to influence airway epithelia and prevent airway remodeling in a asthma mouse model [47].

### TSLP-induced cellular senescence and airway remodeling

TSLP is considered a pivotal cytokine linking innate and adaptive immune disorders [48–50]. Environmental pollutants, including ambient particulate matter, diesel exhaust particles and tobacco smoke, upregulate TSLP expression in airway epithelial cells [51–53]. The TSLP-induced signaling pathway in epithelium has been demonstrated previously [54]. TSLP can induce multiple signaling pathways in asthma, including STAT6, IL-4 [55], IL-1β and TNFα [56], p38 and Jun kinase (JNK ). The central role of Stat3/5 in TSLP-signaling pathway has also been unveiled [57]. Here we further explored the signaling pathway in TSLP-induced airway remodeling in asthma. We found TSLP activates cellular senescent signaling pathways (including the p21 and p16 pathways) to activate airway remodeling *in vivo* and *in vitro*. 

 Currently, TSLP-targeted therapies are being explored in asthma. One recent report demonstrates that neutralization of TSLP can inhibit airway remodeling in a murine model of allergic asthma induced by house dust mites [37]. Antibodies AMG157 targeting TSLP are currently being evaluated for patients with mild atopic asthma in a Phase Ib clinical trial (ClinicalTrials.gov identifier: NCT01405963). However, other small molecules targeting TSLP deserve to be further studied. 

### Stat3-targeted therapy in asthma

Targeted therapy has been widely explored and used in asthma. The use of biologics in asthma, includes the IgE-targeted antibody omalizumab, the IL‑5-targeted antibody mepolizumab, the IL -13-targeted antibody tralokinumab, the IL- 9-targeted monoclonal antibody MEDI-528 and TNFα-blocking antibodies such as infliximab, golimumab and soluble TNFα receptor fusion proteins [58]. Here we found that inhibiting senescent signaling pathways overcome TSLP-induced airway remodeling (Figure 3 & 5). In addition, we found that inhibiting Stat3 by WP1066 can inhibit airway remodeling (Figure 4). 

Previous reports have demonstrated the central role of Stat3 signaling in TSLP-regulated inflammation and airway remodeling in asthma [24]. Specifically, TSLP receptor (TSLPR) activation in bronchial epithelial cells involves Stat3 phosphorylation and induces IL-13 production and cell proliferation [59]. Furthermore, we demonstrated that signaling pathway of Stat3 mediated TSLP-induced airway remodeling in asthma [24]. 

Small molecules that target Stat3 are well established [60]. Several small molecules targeting Stat3 are currently being evaluated for head and neck tumors in Phase I/II clinical trials (clinicaltrials.gov), including WP1066. WP1066 is a cell-permeable, AG 490 tyrphostin analog that effectively inhibits the Jak2-Stat3 pathway [61] and subsequently inhibits the growth of multiple kinds of cells [61–63]. Here we demonstrate that WP1066 targets Stat3 to inhibit airway remodeling *in vitro* and *in vivo* (Figure 4, 5) and this inhibition is mediated by inhibiting the senescent p21 and p16 signaling pathways. Furthermore, we found WP1066 treatment can overcome AHR in an asthma mouse model (Figure 5A ). AHR is a useful marker of airway abnormality in asthma and has been used to predict the course of asthma [64]. These data confirm the effects of Stat3 targeted therapy in asthma and encourage clinical studies to evaluate the therapeutic potential of Stat3-targeted therapy in asthma and the development of other small molecules that target Stat3. However, it is a concern that inhibiting senescent signaling promotes carcinogenesis. It will be important to evaluate the effectiveness and carcinogenesis of any new senescence pathway inhibitor. 

## References

[1] Ramos-Barbon D, Parra-Arrondo A (2011) [Inflammation and remodeling of the distal airways: studies in humans and experimental models]. Arch Bronconeumol: 2047 Suppl 2012:2012-2019. 10.1016/S0300-2896(11)70014-X 21640278

[2] Mauad T, Bel EH, Sterk PJ (2007) Asthma therapy and airway remodeling. J Allergy Clin Immunol 120(5): 997-1009. doi:10.1016/j.jaci.2007.06.031. PubMed: 17681364. 17681364

[3] Beckett PA, Howarth PH (2003) Pharmacotherapy and airway remodelling in asthma? Thorax 58(2): 163-174. doi:10.1136/thorax.58.2.163. PubMed: 12554904. 12554904 PMC1746582

[4] Halwani R, Al-Muhsen S, Hamid Q (2010) Airway remodeling in asthma. Curr Opin Pharmacol 10(3): 236-245. doi:10.1016/j.coph.2010.06.004. PubMed: 20591736. 20591736

[5] Burrows B, Martinez FD, Halonen M, Barbee RA, Cline MG (1989) Association of asthma with serum IgE levels and skin-test reactivity to allergens. N Engl J Med 320(5): 271-277. doi:10.1056/NEJM198902023200502. PubMed: 2911321. 2911321

[6] Tagaya E, Tamaoki J (2007) Mechanisms of airway remodeling in asthma. Allergol Int 56(4): 331-340. doi:10.2332/allergolint.R-07-152. PubMed: 17965576. 17965576

[7] Leonard WJ (2002) TSLP: finally in the limelight. Nat Immunol 3(7): 605-607. doi:10.1038/ni0702-605. PubMed: 12087416. 12087416

[8] Shikotra A, Choy DF, Ohri CM, Doran E, Butler C et al. (2012) Increased expression of immunoreactive thymic stromal lymphopoietin in patients with severe asthma. J Allergy Clin Immunol 129(1): 104-111. doi:10.1016/j.jaci.2011.08.031. PubMed: 21975173. 21975173

[9] Harada M, Hirota T, Jodo AI, Doi S, Kameda M et al. (2009) Functional analysis of the thymic stromal lymphopoietin variants in human bronchial epithelial cells. Am J Respir Cell Mol Biol 40(3): 368-374. doi:10.1165/rcmb.2008-0041OC. PubMed: 18787178. 18787178

[10] Kashyap M, Rochman Y, Spolski R, Samsel L, Leonard WJ (2011) Thymic stromal lymphopoietin is produced by dendritic cells. J Immunol 187(3): 1207-1211. doi:10.4049/jimmunol.1100355. PubMed: 21690322. 21690322 PMC3140600

[11] Ying S, O'Connor B, Ratoff J, Meng Q, Mallett K et al. ( 2005) Thymic stromal lymphopoietin expression is increased in asthmatic airways and correlates with expression of Th2-attracting chemokines and disease severity. J Immunol 174(12): 8183-8190. PubMed: 15944327. 15944327 10.4049/jimmunol.174.12.8183

[12] Ying S, O'Connor B, Ratoff J, Meng Q, Fang C et al. (2008) Expression and cellular provenance of thymic stromal lymphopoietin and chemokines in patients with severe asthma and chronic obstructive pulmonary disease. J Immunol 181(4): 2790-2798. PubMed: 18684970. 18684970 10.4049/jimmunol.181.4.2790

[13] Nguyen KD, Vanichsarn C, Nadeau KC (2010) TSLP directly impairs pulmonary Treg function: association with aberrant tolerogenic immunity in asthmatic airway. Allergy Asthma. Clin Immunol 6(1): 4. 10.1186/1710-1492-6-4 PMC3161393 20230634

[14] Torgerson DG, Ampleford EJ, Chiu GY, Gauderman WJ, Gignoux CR et al. ( 2011) Meta-analysis of genome-wide association studies of asthma in ethnically diverse North American populations. Nat Genet 43(9): 887-892. doi:10.1038/ng.888. PubMed: 21804549. 21804549 PMC3445408

[15] Hirota T, Takahashi A, Kubo M, Tsunoda T, Tomita K et al. (2011) Genome-wide association study identifies three new susceptibility loci for adult asthma in the Japanese population. Nat Genet 43(9): 893-896. doi:10.1038/ng.887. PubMed: 21804548. 21804548 PMC4310726

[16] Boulet LP, Turcotte H, Hudon C, Carrier G, Maltais F (1998) Clinical, physiological and radiological features of asthma with incomplete reversibility of airflow obstruction compared with those of COPD. Can Respir J 5(4): 270-277. PubMed: 9753528. 9753528 10.1155/1998/780739

[17] Fabbri LM, Romagnoli M, Corbetta L, Casoni G, Busljetic K et al. (2003) Differences in airway inflammation in patients with fixed airflow obstruction due to asthma or chronic obstructive pulmonary disease. Am J Respir Crit Care Med 167(3): 418-424. doi:10.1164/rccm.200203-183OC. PubMed: 12426229. 12426229

[18] Aoshiba K, Nagai A (2009) Senescence hypothesis for the pathogenetic mechanism of chronic obstructive pulmonary disease. Proc Am Thorac Soc 6(7): 596-601. doi:10.1513/pats.200904-017RM. PubMed: 19934355. 19934355

[19] Nyunoya T, Monick MM, Klingelhutz A, Yarovinsky TO, Cagley JR et al. (2006) Cigarette smoke induces cellular senescence. Am J Respir Cell Mol Biol 35(6): 681-688. doi:10.1165/rcmb.2006-0169OC. PubMed: 16840774. 16840774 PMC2643295

[20] Tsuji T, Aoshiba K, Nagai A (2004) Cigarette smoke induces senescence in alveolar epithelial cells. Am J Respir Cell Mol Biol 31(6): 643-649. doi:10.1165/rcmb.2003-0290OC. PubMed: 15333326. 15333326

[21] Kuilman T, Michaloglou C, Vredeveld LC, Douma S, van Doorn R et al. (2008) Oncogene-induced senescence relayed by an interleukin-dependent inflammatory network. Cell 133(6): 1019-1031. doi:10.1016/j.cell.2008.03.039. PubMed: 18555778. 18555778

[22] Freund A, Orjalo AV, Desprez PY, Campisi J (2010) Inflammatory networks during cellular senescence: causes and consequences. Trends Mol Med 16(5): 238-246. doi:10.1016/j.molmed.2010.03.003. PubMed: 20444648. 20444648 PMC2879478

[23] Yoshida T, Tuder RM (2007) Pathobiology of cigarette smoke-induced chronic obstructive pulmonary disease. Physiol Rev 87(3): 1047-1082. doi:10.1152/physrev.00048.2006. PubMed: 17615396. 17615396

[24] Wu J, Liu F, Zhao J, Wei Y, Lv J et al. (2012) Thymic stromal lymphopoietin promotes asthmatic airway remodelling in human lung fibroblast cells through STAT3 signalling pathway. Cell Biochem Funct 31(6): 496-503. PubMed: 23192865. 23192865 10.1002/cbf.2926

[25] Yang G, Li Y, Nishimura EK, Xin H, Zhou A et al. (2008) Inhibition of PAX3 by TGF-beta modulates melanocyte viability. Mol Cell 32: 554-563. doi:10.1016/j.molcel.2008.11.002. PubMed: 19026785. 19026785

[26] Camp RL, Rimm EB, Rimm DL (1999) Met expression is associated with poor outcome in patients with axillary lymph node negative breast carcinoma. Cancer 86: 2259-2265. doi:10.1002/(SICI)1097-0142(19991201)86:11. PubMed: 10590366. 10590366

[27] Cao J, Wan L, Hacker E, Lenna S, Jimenez-Cervantes C et al. (2013) MC1R is a potent regulator of PTEN after UV exposure in melanocytes. Mol Cell (. (2013)) PubMed: 23973372. 10.1016/j.molcel.2013.08.010 PMC3792490 23973372

[28] Kim SJ, Shin JH, Kim SC, Park CK, Kim SW (2013) Preventive effects of oral tolerance on allergic inflammation and airway remodeling in a murine model. Am J Rhinol Allergy 27(1): e11-e16. doi:10.2500/ajra.2013.27.3853. PubMed: 23406589. 23406589

[29] Ramakrishna G, Anwar T, Angara RK, Chatterjee N, Kiran S et al. (2012) Role of cellular senescence in hepatic wound healing and carcinogenesis. Eur J Cell Biol 91(10): 739-747. doi:10.1016/j.ejcb.2012.08.002. PubMed: 22980320. 22980320

[30] Lanigan F, Geraghty JG, Bracken AP (2011) Transcriptional regulation of cellular senescence. Oncogene 30(26): 2901-2911. doi:10.1038/onc.2011.34. PubMed: 21383691. 21383691

[31] Tuder RM, Yoshida T (2011) Stress responses affecting homeostasis of the alveolar capillary unit. Proc Am Thorac Soc 8(6): 485-491. doi:10.1513/pats.201103-029MW. PubMed: 22052924. 22052924 PMC3359075

[32] Campisi J, d'Adda di Fagagna F (2007) Cellular senescence: when bad things happen to good cells. Nat Rev Mol Cell Biol 8(9): 729-740. doi:10.1038/nrm2233. PubMed: 17667954. 17667954

[33] Semlali A, Jacques E, Rouabhia M, Milot J, Laviolette M et al. (2010) Regulation of epithelial cell proliferation by bronchial fibroblasts obtained from mild asthmatic subjects. Allergy 65(11): 1438-1445. doi:10.1111/j.1398-9995.2010.02376.x. PubMed: 20456314. 20456314

[34] Damera G, Fogle HW, Lim P, Goncharova EA, Zhao H et al. (2009) Vitamin D inhibits growth of human airway smooth muscle cells through growth factor-induced phosphorylation of retinoblastoma protein and checkpoint kinase 1. Br J Pharmacol 158(6): 1429-1441. doi:10.1111/j.1476-5381.2009.00428.x. PubMed: 19814732. 19814732 PMC2795210

[35] Fedorov IA, Wilson SJ, Davies DE, Holgate ST (2005) Epithelial stress and structural remodelling in childhood asthma. Thorax 60(5): 389-394. doi:10.1136/thx.2004.030262. PubMed: 15860714. 15860714 PMC1758889

[36] Chuang CY, Chang CH, Huang YL (2009) Thioredoxin mediates remodeling factors of human bronchial epithelial cells upon interaction with house dust mite-stimulated eosinophils. Inhal Toxicol 21(2): 153-167. doi:10.1080/08958370802368730. PubMed: 18800270. 18800270

[37] Chen ZG, Zhang TT, Li HT, Chen FH, Zou XL et al. (2013) Neutralization of TSLP inhibits airway remodeling in a murine model of allergic asthma induced by chronic exposure to house dust mite. PLOS ONE 8(1): e51268. doi:10.1371/journal.pone.0051268. PubMed: 23300949. 23300949 PMC3534685

[38] Reddel RR, Ke Y, Kaighn ME, Malan-Shibley L, Lechner JF et al. (1988) Human bronchial epithelial cells neoplastically transformed by v-Ki-ras: altered response to inducers of terminal squamous differentiation. Oncogene Res 3(4): 401-408. PubMed: 3067190. 3067190

[39] Dimri GP, Lee X, Basile G, Acosta M, Scott G et al. (1995) A biomarker that identifies senescent human cells in culture and in aging skin in vivo. Proc Natl Acad Sci U S A 92: 9363-9367. doi:10.1073/pnas.92.20.9363. PubMed: 7568133. 7568133 PMC40985

[40] Yao W, Zhang Y, Jabeen R, Nguyen ET, Wilkes DS et al. (2013) Interleukin-9 is required for allergic airway inflammation mediated by the cytokine TSLP. Immunity 38: 360-372. doi:10.1016/j.immuni.2013.01.007. PubMed: 23376058. 23376058 PMC3582776

[41] Ota K, Dohi Y, Brydun A, Nakanome A, Ito S et al. (2011) Identification of senescence-associated genes and their networks under oxidative stress by the analysis of Bach1. Antioxid Redox Signal 14(12): 2441-2451. doi:10.1089/ars.2010.3574. PubMed: 21110788. 21110788

[42] Collado M, Serrano M (2010) Senescence in tumours: evidence from mice and humans. Nat Rev Cancer 10(1): 51-57. doi:10.1038/nrc2772. PubMed: 20029423. 20029423 PMC3672965

[43] McDonald ER 3rd, El-Deiry WS (2001) Checkpoint genes in cancer. Ann Med 33(2): 113-122. doi:10.3109/07853890109002066. PubMed: 11327114. 11327114

[44] Tuder RM, Kern JA, Miller YE (2012) Senescence in chronic obstructive pulmonary disease. Proc Am Thorac Soc 9(2): 62-63. doi:10.1513/pats.201201-012MS. PubMed: 22550244. 22550244 PMC3359109

[45] Shivshankar P, Brampton C, Miyasato S, Kasper M, Thannickal VJ et al. (2012) Caveolin-1 deficiency protects from pulmonary fibrosis by modulating epithelial cell senescence in mice. Am J Respir Cell Mol Biol 47(1): 28-36. doi:10.1165/rcmb.2011-0349OC. PubMed: 22362388. 22362388 PMC3402795

[46] Burns TF, Dobromilskaya I, Murphy SC, Gajula RP, Thiyagarajan S et al. (2013) Inhibition of TWIST1 Leads to Activation of Oncogene-Induced Senescence in Oncogene Driven Non-Small Cell Lung Cancer. Mol Cancer Res 11(4): 329-338. doi:10.1158/1541-7786.MCR-12-0456. PubMed: 23364532. 23364532 PMC3631276

[47] Chuang CY, Chang CH, Huang YL (2009) Thioredoxin mediates remodeling factors of human bronchial epithelial cells upon interaction with house dust mite-stimulated eosinophils. Inhal Toxicol 21(2): 153-167. doi:10.1080/08958370802368730. PubMed: 18800270. 18800270

[48] Liu YJ, Soumelis V, Watanabe N, Ito T, Wang YH et al. (2007) TSLP: an epithelial cell cytokine that regulates T cell differentiation by conditioning dendritic cell maturation. Annu Rev Immunol 25: 193-219. doi:10.1146/annurev.immunol.25.022106.141718. PubMed: 17129180. 17129180

[49] Liu YJ (2009) TSLP in epithelial cell and dendritic cell cross talk. Adv Immunol 101: 1-25. doi:10.1016/S0065-2776(08)01001-8. PubMed: 19231591. 19231591 PMC3645262

[50] Soumelis V (2012) TSLP: from allergy to vaccine adjuvant. Eur J Immunol 42(2): 293-295. doi:10.1002/eji.201142337. PubMed: 22266716. 22266716

[51] Bleck B, Tse DB, Curotto de Lafaille MA, Zhang F, Reibman J (2008) Diesel exhaust particle-exposed human bronchial epithelial cells induce dendritic cell maturation and polarization via thymic stromal lymphopoietin. J Clin Immunol 28(2): 147-156. doi:10.1007/s10875-007-9149-0. PubMed: 18049884. 18049884 PMC2757761

[52] Bleck B, Tse DB, Gordon T, Ahsan MR, Reibman J (2010) Diesel exhaust particle-treated human bronchial epithelial cells upregulate Jagged-1 and OX40 ligand in myeloid dendritic cells via thymic stromal lymphopoietin. J Immunol 185(11): 6636-6645. doi:10.4049/jimmunol.1000719. PubMed: 20974985. 20974985 PMC3927452

[53] Nakamura Y, Miyata M, Ohba T, Ando T, Hatsushika K et al. (2008) Cigarette smoke extract induces thymic stromal lymphopoietin expression, leading to T(H)2-type immune responses and airway inflammation. J Allergy Clin Immunol 122(6): 1208-1214. doi:10.1016/j.jaci.2008.09.022. PubMed: 18926564. 18926564

[54] Rochman Y, Leonard WJ (2008) Thymic stromal lymphopoietin: a new cytokine in asthma. Curr Opin Pharmacol 8: 249-254. doi:10.1016/j.coph.2008.03.002. PubMed: 18450510. 18450510 PMC2518061

[55] Kato A, Favoreto S Jr, Avila PC, Schleimer RP (2007) TLR3- and Th2 cytokine-dependent production of thymic stromal lymphopoietin in human airway epithelial cells. J Immunol 179(2): 1080-1087. PubMed: 17617600. 17617600 10.4049/jimmunol.179.2.1080 PMC2220044

[56] Allakhverdi Z, Comeau MR, Jessup HK, Yoon BR, Brewer A et al. (2007) Thymic stromal lymphopoietin is released by human epithelial cells in response to microbes, trauma, or inflammation and potently activates mast cells. J Exp Med 204(2): 253-258. doi:10.1084/jem.20062211. PubMed: 17242164. 17242164 PMC2118732

[57] Liu W, Xu LS, Liu QJ, Dong FZ, Qiu RF et al. (2012) Two single nucleotide polymorphisms in TSLP gene are associated with asthma susceptibility in Chinese Han population. Exp Lung Res 38(8): 375-382. doi:10.3109/01902148.2012.714840. PubMed: 22913730. 22913730

[58] Pelaia G, Vatrella A, Maselli R (2012) The potential of biologics for the treatment of asthma. Nat Rev Drug Discov 11(12): 958-972. doi:10.1038/nrd3792. PubMed: 23197041. 23197041

[59] Semlali A, Jacques E, Koussih L, Gounni AS, Chakir J (2010) Thymic stromal lymphopoietin-induced human asthmatic airway epithelial cell proliferation through an IL-13-dependent pathway. J Allergy Clin Immunol 125(4): 844-850. doi:10.1016/j.jaci.2010.01.044. PubMed: 20236697. 20236697

[60] Levy DE, Inghirami G (2006) STAT3: a multifaceted oncogene. Proc Natl Acad Sci U S A 103(27): 10151-10152. doi:10.1073/pnas.0604042103. PubMed: 16801534. 16801534 PMC1502425

[61] Hussain SF, Kong LY, Jordan J, Conrad C, Madden T et al. (2007) A novel small molecule inhibitor of signal transducers and activators of transcription 3 reverses immune tolerance in malignant glioma patients. Cancer Res 67(20): 9630-9636. doi:10.1158/0008-5472.CAN-07-1243. PubMed: 17942891. 17942891

[62] Ferrajoli A, Faderl S, Van Q, Koch P, Harris D et al. (2007) WP1066 disrupts Janus kinase-2 and induces caspase-dependent apoptosis in acute myelogenous leukemia cells. Cancer Res 67(23): 11291-11299. doi:10.1158/0008-5472.CAN-07-0593. PubMed: 18056455. 18056455

[63] Kong LY, Abou-Ghazal MK, Wei J, Chakraborty A, Sun W et al. (2008) A novel inhibitor of signal transducers and activators of transcription 3 activation is efficacious against established central nervous system melanoma and inhibits regulatory T cells. Clin Cancer Res 14(18): 5759-5768. doi:10.1158/1078-0432.CCR-08-0377. PubMed: 18794085. 18794085 PMC2583362

[64] James AL, Wenzel S (2007) Clinical relevance of airway remodelling in airway diseases. Eur Respir J 30(1): 134-155. doi:10.1183/09031936.00146905. PubMed: 17601971. 17601971

